# Exposure time, running and skill-related performance in international u20 rugby union players during an intensified tournament

**DOI:** 10.1371/journal.pone.0186874

**Published:** 2017-11-14

**Authors:** Christopher J. Carling, Mathieu Lacome, Eamon Flanagan, Pearse O’Doherty, Julien Piscione

**Affiliations:** 1 Institute of Coaching and Performance, University of Central Lancashire, Preston, United Kingdom; 2 Research Department, French Rugby Union, Marcoussis, France; 3 Irish Rugby Football Union, Fitness Department, Dublin, Ireland; 4 Statsports Technologies^™^, Newry, Northern Ireland; Universidade de Tras-os-Montes e Alto Douro, PORTUGAL

## Abstract

**Purpose:**

This study investigated exposure time, running and skill-related performance in two international u20 rugby union teams during an intensified tournament: the 2015 Junior World Rugby Championship.

**Method:**

Both teams played 5 matches in 19 days. Analyses were conducted using global positioning system (GPS) tracking (Viper 2^™^, Statsports Technologies Ltd) and event coding (Opta Pro^®^).

**Results:**

Of the 62 players monitored, 36 (57.1%) participated in 4 matches and 23 (36.5%) in all 5 matches while player availability for selection was 88%. Analyses of team running output (all players completing >60-min play) showed that the total and peak 5-minute high metabolic load distances covered were likely-to-very likely moderately higher in the final match compared to matches 1 and 2 in back and forward players. In individual players with the highest match-play exposure (participation in >75% of total competition playing time and >75-min in each of the final 3 matches), comparisons of performance in matches 4 and 5 versus match 3 (three most important matches) reported moderate-to-large decreases in total and high metabolic load distance in backs while similar magnitude reductions occurred in high-speed distance in forwards. In contrast, skill-related performance was unchanged, albeit with trivial and unclear changes, while there were no alterations in either total or high-speed running distance covered at the end of matches.

**Conclusions:**

These findings suggest that despite high availability for selection, players were not over-exposed to match-play during an intensified u20 international tournament. They also imply that the teams coped with the running and skill-related demands. Similarly, individual players with the highest exposure to match-play were also able to maintain skill-related performance and end-match running output (despite an overall reduction in the latter). These results support the need for player rotation and monitoring of performance, recovery and intervention strategies during intensified tournaments.

## Introduction

Rugby union is an intermittent team sport requiring players to repeatedly perform bouts of high-speed running interspersed with periods of low-speed activity [1]. Intense static exertions such as scrummaging, physical collisions and tackles also occur frequently throughout play [2]. On average, forward and back players at elite senior levels are shown to spend 14% and 8% of their match time in highly intense activities such as sprinting and tackling and in scrums, rucks and mauls [3]. Combined, these physical demands are shown to result in high levels of muscle damage [4,5], neuromuscular and perceptual fatigue [6] and compromised immunity [7] post-competition. While generally transient in nature, such disturbances typically persist for 24–48 h following match-play although muscle damage can last for several days with large variations in recovery kinetics reported across individuals [8]. At elite senior levels however, a single match is generally played per week over the course of the season [9]. Therefore, the time interval separating consecutive matches is sufficient in theory to ensure complete physical and physiological recovery [10].

In contrast to elite senior rugby union competition, congested competition schedules involving multiple matches played in a short time period occur in players in elite junior categories. For example, the annual World Rugby u20 World Cup schedule requires national teams to participate in 5 matches over a 19-day period. If recovery time between successive matches is short, residual fatigue, muscle damage and reduced immunity have the potential to compromise ensuing match performance [11]. Yet to our knowledge, no data currently exist on the potential effects on match performance (e.g., running, technical actions) of participation in intensified tournaments such as the u20 World Cup. Related research in junior Rugby League players has reported a progressive accumulation of fatigue represented by a reduced capacity to perform high-speed exercise during tournaments where multiple matches were played over a 5-day period [12]. An investigation more representative of the u20 World Cup schedule (cycle of 4 matches in 22 days vs. 5 matches in 19 days), albeit in professional rugby league players demonstrated fluctuations in running activity with reductions in high-speed and increases in low-speed distance covered in the latter matches [13]. No information was reported on any potential changes in technical skill-related performance in either study. Thus research investigating match-to-match running and technical skill-related performance during the u20 World Cup is warranted.

Despite the aforementioned potential risk of fatigue accumulation and compromised competitive performance associated with insufficient recovery time during intensified tournaments, no information exists on the actual exposure time of players to match-play. Recent research in a professional association football club [14] has shown that despite the frequent occurrence of periods of match congestion across the season, squad rotation strategies were employed by the coaching staff to ensure that players did not endure over-exposure. Thus, in our opinion, similar data across tournaments such as the u20 World Cup are necessary to determine the actual extent of player exposure and therefore the aforementioned potential risk of compromised match performance.

This study examined exposure time and the effects of an intensified tournament on running and skill-related match performance in international u20 players during the 2015 World Rugby u20 World Cup.

## Materials and methods

### Experimental approach to the problem

The present study was conducted during the 2015 World Rugby u20 World Cup tournament. Participation time for each player was recorded to determine the extent of match exposure over this intensified schedule. Global positioning systems (GPS) and match analysis software were used to gather data related to match running and skill performance and examine the potential effects of the congested schedule on performance notably in players with high exposure time to match-play.

### Participants

All players were members of the French or Irish national u20 teams. Altogether, 63 players (age: 19.8 ± 0.5 y, body mass: 99.1 ± 9.1 kg, stature: 185.4 ± 7.0 cm) participated. Prior to participation, all players received comprehensive verbal and written explanations of the study and provided voluntarily signed informed consent to wear GPS in competitive matches and to participate in the collection of performance data for the entirety of the Championship. These data arose as a condition of selection for their national team in which player performance was routinely measured over the course of the competitive season [15]. Nevertheless, institutional board approval for the study was obtained from the Medical Council of the Federation Française de Rugby. To ensure confidentiality, all performance data were anonymized. This study conformed to the recommendations of the Declaration of Helsinki.

### Competition

During the competition, each team played 5 matches in 19 days. A total of 4 days (94-98h) separated matches 1 and 2 and matches 2 and 3 and 5 days (118-120h) separated matches 3 and 4 and matches 4 and 5. Altogether, 226 match observations (forwards = 128 and backs = 98 matches) were collected. All participating players followed standardized recovery protocols over the course of the competition: consumption of a minimum of 40 g carbohydrates and 20 g protein in liquid or whole food form immediately after competition. Players were also requested to use cold bath, massages and compression garments. The day following the match, players performed recovery protocols (hydrotherapy session, foam rolls, compression garments) and received appropriate nutritional and hydration plans.

### Study design

In order to conduct the analyses, two categories of performance measures were employed:

#### 1. Time-motion analyses of running performance

Each player wore a 10-Hz GPS unit (mass = 50g, size = 86x33x20mm; Viper 2^™^, Statsports Technologies^™^, Newry, Northern Ireland) in a bespoke pocket fitted in their playing jersey which positioned the GPS unit on the upper thoracic spine between the scapulae. Independent testing has reported low typical error of measurement (range: 0.7–1.7%) and coefficient of variation (2.0–2.9%) as well as low absolute error (2.9–3.0%) over a range of activities including repeated 30m shuttle runs, a 132.3m circuit simulating soccer activity and 16-minute duration small-sided matches (unpublished data, Marathon Performance Center, 2014). All participants were familiarised with the devices as part of their daily training and practice in the season leading up to the 2015 World Rugby u20 World Cup.

The GPS units were turned on at least 30 minutes prior to each match to facilitate satellite signal connection. Information on the average number of satellites to which GPS devices were connected and values for the horizontal dilution of precision were unavailable. Following the matches, GPS data were downloaded to a laptop and analysed with proprietary software (STATSport Viper Rugby v2.6.1.173, STATSports Technologies Ltd., Ireland, UK). Players whose GPS unit suffered a loss of signal for a period of time within the match were excluded (i.e., GPS fell on the ground, spikes in the data, n = 11). Each file was cropped to ensure that only data recorded when the player was on the field was included. A number of locomotor variables were analysed: total distance run (TD) and that covered at high-running speeds (HS) (threshold > 5.5 m.s^-1^). High-metabolic load distance (HI distance + distance covered while accelerating above 2 m.s^-2^) [16] and the total number of high-speed activities (> 5.5 m.s^-1^) and accelerations (> 2 m.s^-2^) were also recorded. Finally, the peak 5-min of HMLD (HMLD.Peak5min) was recorded for each match and player using a 5-min rolling average with step 0.1-s.

#### 2. Match analyses of skill-related performance

Measures of skill-related performance defined by Opta Pro^®^ data provider and coded by the company’s match analysts using the Sportscode software (Sportstec, Australia) included the total number of tackles, passes and carries along with respective completion rates in these events. Effective playing time (time the ball was in play) was also recorded. Although no data exists for elite rugby union, high levels of Opta inter-operator reliability for coding match events in elite association football have been demonstrated [17].

### Data collection procedures

#### 1. Participation patterns

Exposure time was recorded for each individual player. Basic metrics quantified from this data included total number of and percentage of the players completing: (1) 3, 4 and 5 matches respectively, (2) 3, 4 and 5 matches played successively, (3) at least 60-min play [18] in 3, 4 and 5 matches played successively, (4) >240-min (equivalent to 3 complete matches) and >320-min (equivalent to 4 complete matches) total participation time over the tournament. Time loss injuries and subsequent unavailability for match selection were prospectively recorded by the team physicians respective to both teams.

#### 2. Overall team running and skill-related performance

To investigate accumulated changes in overall team performance, running and skill-related performance measures were normalised relative to each player’s participation time and compared across matches 1 to 5. Players competing for <60-min were excluded. A total of 171 match observations were collected including 77 and 94 observations for backs and forwards respectively.

#### 3. Running and skill-related performance in “high exposure” players

Players with high exposure time notably during the final three matches were assessed separately. These three matches were selected as these were considered to be the highest standard and most important matches of the competition (e.g., semi-finals, finals or matches to determine team seeding in the following year’s u20 world cup) and for which coaching staff habitually select their best performers. Hence players should have been subjected to the highest physical and technical demands in these three matches. Inclusion criteria were: (1) participation in at least 75-min in each of the final 3 matches in the series, and (2) played more than 320-min over the course of the competition (>75% of total playing time).

To investigate potential accumulated changes in individual match-performance, the aforementioned running and skill-related measures were normalised relative to each player’s total playing time and compared from Match 3 to Match 5. The total distance and high metabolic load distance covered were also compared for the final 10-min period versus the mean value (minus first and last 10-min periods) for the other 10-min periods.

### Statistical analysis

Statistical analyses were performed using R statistical software (R. 3.1.0, R Foundation for Statistical Computing) using the *lme4* and *psychometric* package. Means and standard deviations for each group or playing time were derived from a *generalized linear model*, with the distribution and link function contingent upon the nature of the dependent variable. The overdispersed Poisson distribution was chosen for modelling the data from the match analyses and the normal distribution was chosen for distances from the time-motion analyses. For each analysis, the match (Match 1 to Match 5) was included as a fixed effect while players and teams were included as random effects. The % differences between mean values with 90% confidence intervals (CI) are reported.

A magnitude-based inferential approach was adopted [19,20]. Effect sizes (ES) were quantified to indicate the practical meaningfulness of the differences in mean values. Standardisation was performed with the estimated marginal means and associated variance provided by the *generalized linear model*. The ES was classified as trivial (0–0.19), small (0.20–0.59), moderate (0.6–1.19), large (1.20–1.99) and very large (>2.0). If the 90% CI over-lapped small positive and negative values, the magnitude was deemed unclear. The chances that the changes in running- or skill-related performance were greater for a group (i.e., greater than the smallest worthwhile change, SWC (0.2 multiplied by the between-subject standard deviation, based on Cohen’s *d* principle)), similar or smaller than the other group were calculated. Quantitative chances of greater or smaller changes in performance variable were assessed qualitatively with the following scale: 25−75%, possible; 75−95%, likely; 95−99%, very likely; >99%, almost certain. [21]

## Results

### Match exposure

The patterns of participation of players and exposure to periods of match congestion cycles are presented in Table 1. Of the 62 players, 36 (57%) played 4 matches and 23 (37) played 5 matches. Of these appearances, 39, 28 and 23 players played 3, 4 and 5 matches successively (62, 44, and 37% respectively). The proportion of backs and forwards who played 3, 4 and 5 matches successively with over 60-min of playing time was 14, 6 and 6% respectively for forwards and 35, 19 and 8% respectively for backs. Player availability for selection overall across the competition was 88%.

**Table 1:** Overall participation of players in the competition and exposure to match congestion cycles.

Match exposure	*ALL PLAYERS (62)*		*FORWARDS (36)*		*BACKS (26)*	
	Occurrences (n)	Relative Nb (%)	Occurrences (n)	Relative Nb (%)	Occurrences (n)	Relative Nb (%)
***Matches played***						
*Played >320 min in total*	14	22%	5	14%	9	35%
*Played >240 min in total*	23	37%	10	28%	13	50%
*Participations in 3 games (nb)*	47	75%	26	72%	21	81%
*Participations in 4 games (nb)*	36	57%	19	53%	17	65%
*Participations in 5 games (nb)*	23	37%	13	36%	10	38%
**Multiple match cycles**						
*Participations in 3 successive games (nb)*	39	62%	23	64%	16	62%
*Participations in 3 successive games >60-min (nb)*	14	22%	5	14%	9	35%
*Participations in 4 successive games (nb)*	28	44%	16	44%	12	46%
*Participations in 4 successive games >60-min (nb)*	7	11%	2	6%	5	19%
*Participations in 5 successive games (nb)*	23	37%	13	36%	10	38%
*Participations in 5 successive games >60-min (nb)*	4	6%	2	6%	2	8%

Nb: Number.

### Overall team match performance

**Fig 1:**
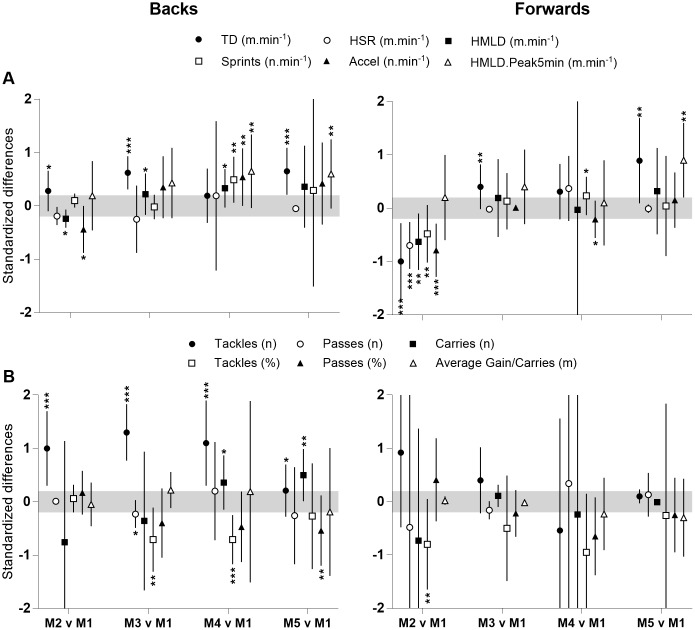
Standardised differences in running (panel A)- and skill (panel B)- related performance between match 1 and match 2 to 5 in forwards and backs. Grey zone stands for trivial zone (effect size ± 0.2). TD: Total distance; HSR (High speed running); HMLD: High metabolic load distance. Accel: Accelerations; HMLD.Peak5min: Peak 5-min of high metabolic load distance.

Table 2 reports running and skill-related performance of players completing at least 60-min in the matches while Fig 1 reports standardised changes in running and skill-related performance in match 2 to 5 compared with match 1. Overall, unclear to likely small changes in HSR, HMLD, sprints and accelerations were observed for backs (ES: -0.44 ±0.44 to 0.54 ±0.54) between match 1 and the other matches. Regarding total distance covered, moderate increases were observed for match 3 and 5 compared with match 1 (ES: 0.62 ±0.31 and 0.65 ±0.44 respectively). In forwards, unclear to small changes were reported in all running-performance variables except for total distance covered. Regarding total distance covered, small to moderate increases were observed between match 3 and 5 compared to match 1 (ES: 0.40 ±0.42 and 0.89 ±0.80 respectively).

**Table 2:** Running and skill- performance in players competing at least 60-min from match 1 to match 5.

**BACKS**	Match 1 (13)	Match 2 (14)	Match 3 (13)	Match 4 (12)	Match 5 (12)
TD (m.min^-1^)	66.8 ± 6.0	64.7 ± 8.3	71.3 ± 7.9	68.0 ± 5.1	70.3 ± 3.6
HSR (m.min^-1^)	4.4 ± 2.0	4.1 ± 1.4	4.0 ± 1.5	4.9 ± 2.3	4.4 ± 1.8
HMLD (m.min^-1^)	10.5 ± 2.0	10.0 ± 1.9	11.1 ± 2.8	11.3 ± 2.4	11.3 ± 2.0
Sprints (n.min^-1^)	0.24 ± 0.08	0.25 ± 0.07	0.24 ± 0.08	0.29 ± 0.1	0.27 ± 0.08
Accel (n.min^-1^)	0.31 ± 0.08	0.27 ± 0.09	0.34 ± 0.13	0.36 ± 0.11	0.35 ± 0.11
HMLD.Peak5min (m.min^-1^)	25.7 ± 5.2	26.9 ± 6.0	28.3 ± 6.0	30.6 ± 9.0	30.0 ± 8.3
Tackles (n)	0.05 ± 0.03	0.09 ± 0.04	0.10 ± 0.04	0.08 ± 0.03	0.06 ± 0.03
Passes (n)	0.10 ± 0.12	0.10 ± 0.11	0.07 ± 0.09	0.12 ± 0.1	0.07 ± 0.04
Carries (n)	0.08 ± 0.04	0.05 ± 0.03	0.06 ± 0.04	0.09 ± 0.03	0.10 ± 0.06
Tackles (%)	0.82 ± 0.30	0.83 ± 0.15	0.62 ± 0.23	0.63 ± 0.18	0.74 ± 0.29
Passes (%)	0.97 ± 0.06	0.98 ± 0.05	0.93 ± 0.11	0.93 ± 0.12	0.91 ± 0.14
Average Gain/Carries (m)	5.23 ± 3.00	5.10 ± 2.28	6.59 ± 8.05	5.82 ± 2.89	4.70 ± 2.29
**FORWARDS**	Match 1 (12)	Match 2 (10)	Match 3 (12)	Match 4 (9)	Match 5 (10)
TD (m.min^-1^)	59.8 ± 4.7	53.8 ± 6.4	62.7 ± 8.2	61.3 ± 4.6	63.6 ± 3.5
HSR (m.min^-1^)	1.1 ± 0.8	0.6 ± 0.5	1.0 ± 0.8	1.4 ± 0.7	1.1 ± 0.8
HMLD (m.min^-1^)	6.5 ± 2.2	5.2 ± 1.9	7.0 ± 2.4	6.4 ± 3.0	7.2 ± 2.0
Sprints (n.min^-1^)	0.09 ± 0.06	0.06 ± 0.05	0.10 ± 0.07	0.10 ± 0.06	0.09 ± 0.06
Accel (n.min^-1^)	0.31 ± 0.16	0.19 ± 0.11	0.31 ± 0.16	0.27 ± 0.16	0.33 ± 0.11
HMLD.Peak5min (m.min^-1^)	15.4 ± 3.2	16.3 ± 5.4	17.4 ± 5.1	15.7 ± 6.5	19.3 ± 4.9
Tackles (n)	0.11 ± 0.06	0.15 ± 0.01	0.13 ± 0.05	0.08 ± 0.03	0.12 ± 0.06
Passes (n)	0.03 ± 0.04	0.01 ± 0.02	0.02 ± 0.03	0.04 ± 0.04	0.04 ± 0.06
Carries (n)	0.09 ± 0.06	0.05 ± 0.05	0.10 ± 0.08	0.08 ± 0.06	0.09 ± 0.06
Tackles (%)	0.96 ± 0.06	0.89 ± 0.11	0.92 ± 0.09	0.83 ± 0.19	0.94 ± 0.08
Passes (%)	0.99 ± 0.03	1.00 ± 0.00	0.98 ± 0.06	0.91 ± 0.19	0.98 ± 0.06
Average Gain/Carries (m)	1.91 ± 1.68	1.93 ± 1.51	1.88 ± 1.34	1.56 ± 1.04	1.46 ± 1.11
*Effective playing time (min)*	29.3 ± 2.1	29.6 ± 3.4	35.3 ± 8.5	32.2 ± 0.2	33.1 ± 2.5

TD: Total distance; HSR: High speed running; HMLD: High metabolic load distance; ES: Effect size; % chances: % chances that the true difference is +ive/ trivial/ -ive.

Number in parenthesis refers to the number of players analysed.

Regarding skill-related performance, unclear to small differences in the frequency of passes and carries were observed between match 1 and the other matches. Likely moderate increases were reported between the frequencies of tackles performed by backs between match 1 and matches 2 to 4 (ES: 1.00 ±0.70; 1.30 ±0.53 and 1.10 ±0.80 for matches 2, 3 and 4 respectively). Unclear to small fluctuations in pass success rates or average gain per carries were observed in backs. Likely moderate decreases in tackles success rates occurred between match 1 and match 3 and 4 in backs (ES: 1.30 ±0.53 and 1.10 ±0.80). In forwards, there were unclear to trivial effect size differences in the frequency and success rates of skill-related performance measures between match 1 and the other matches except in tackling actions for which there was a moderate decrease in match 1 vs match 2 and a moderate decrease in passing success rates in match 1 vs match 4.

There was no difference in effective playing time between Match 1 and 2 but possibly moderate to likely large increases were observed in Match 3, 4 and 5 compared with Match 1 (ES: 0.90 ±0.49; 1.75 ±0.51; 1.43 ±0.50 respectively).

### Match performance in “high exposure” players

Table 3 reports running and skill-related performance in high exposure players. In backs, likely moderate to large decreases in total distance covered and HLMD distance covered were reported between match 3 versus match 4 and 5 (ES: -0.61 ±0.78 to -1.70 ±1.50). Regarding HSR distance covered as well as sprints and acceleration frequencies, only unclear differences were reported between match 3 versus match 4 and 5. In forwards, except for HSR distance covered (ES: 1.20 ±0.78 and 0.69 ±0.75 for Match 4 and 5 compared to Match 3 respectively), only unclear differences were reported in running related performance.

**Table 3:** Running and skill-performance in “high-exposure players” from match 3 to match 5.

**BACKS (5)**	Match 3	Match 4	Match 5	Match 3 vs Match 4		Match 3 vs Match 5	
				ES	% chances	ES	% chances
TD (m.min^-1^)	73.7 ± 7.1	66.1 ± 4.6	69.9 ± 3.5	-1.20 ±0.80	0/2/97	-0.61 ±0.78	4/14/81
HSR (m.min^-1^)	4.9 ± 1.0	4.8 ± 1.7	3.8 ± 0.9	-0.05 ±0.79	29/33/37	-0.98 ±1.30	6/9/85
HMLD (m.min^-1^)	12.2 ± 0.6	11.2 ± 1.8	10.8 ± 0.9	-0.67 ±0.81	4/13/83	-1.70 ±1.50	2/3/95
Sprints (n.min^-1^)	0.29 ± 0.02	0.30 ± 0.06	0.26 ± 0.05	0.22 ±8.90	50/3/47	-0.57 ±0.94	9/17/75
Accel (n.min^-1^)	0.40 ± 0.10	0.40 ± 0.12	0.37 ± 0.11	-0.03 ±2.40	44/11/45	-0.30 ±0.96	19/24/57
HMLD.Peak5min (m.min^-1^)	31.8 ± 5.0	28.4 ± 6.3	26.9 ± 1.2	-0.53.± 1.04	12/15/72	-1.20.± 1.04	2/4/94
Tackles (n)	0.12 ± 0.05	0.08 ± 0.03	0.06 ± 0.02	-0.87 ±0.91	3/8/89	-1.58± 1.04	1/1/98
Passes (n)	0.03 ± 0.03	0.06 ± 0.04	0.06 ± 0.05	0.72 ±1.20	79/12/9	0.66 ±1.10	78/14/9
Carries (n)	0.08 ± 0.02	0.07 ± 0.02	0.08 ± 0.05	-0.15 ±1.60	35/18/48	-0.06 ±0.91	31/30/39
Tackles (%)	0.59 ± 0.19	0.59 ± 0.20	0.80 ± 0.12	0.01 ±1.00	37/27/36	1.20 ±0.99	95/4/2
Passes (%)	0.95 ± 0.11	0.86 ± 0.14	0.92 ± 0.14	-0.60 ±1.00	10/15/75	-0.24 ±1.00	22/25/53
Average Gain/Carries (m)	5.52 ± 7.35	5.71 ± 3.09	3.46 ± 1.51	0.03 ±0.88	36/32/32	-0.35 ±0.94	15/23/61
**FORWARDS (5)**	Match 1	Match 2	Match 3	Match 3 vs Match 4		Match 3 vs Match 5	
				ES	% chances	ES	% chances
TD (m.min^-1^)	61.4 ± 8.8	62.5 ± 3.5	64.2 ± 2.5	0.15 ±0.61	44/39/17	0.40 ±0.66	70/24/7
HSR (m.min^-1^)	0.5 ± 0.3	1.4 ± 0.8	1.0 ± 0.8	1.20 ±0.78	98/1/0	0.69 ±0.75	86/11/3
HMLD (m.min^-1^)	5.8 ± 2.0	6.5 ± 3.6	7.1 ± 1.6	0.23 ±0.69	53/32/15	0.65 ±1.10	75/15/10
Sprints (n.min^-1^)	0.06 ± 0.04	0.11 ± 0.07	0.08 ± 0.05	0.91 ±0.80	93/6/1	0.43 ±0.88	67/21/11
Accel (n.min^-1^)	0.28 ± 0.10	0.29 ± 0.19	0.34 ± 0.1	0.07 ±0.85	40/31/30	0.53 ±0.78	76/18/6
HMLD.Peak5min (m.min^-1^)	17.1 ± 6.1	14.6 ± 7.6	18.4 ± 5.6	-0.33.± 1.04	20/20/60	0.19.± 1.04	51/23/27
Tackles (n)	0.13 ± 0.05	0.10 ± 0.02	0.13 ± 0.05	-0.85 ±1.10	6/11/83	0.01 ±1.1	38/25/37
Passes (n)	0.03 ± 0.03	0.06 ± 0.04	0.05 ± 0.07	0.77 ±0.86	87/9/4	0.41 ±0.57	74/22/4
Carries (n)	0.10 ± 0.08	0.09 ± 0.06	0.10 ± 0.06	-0.07 ±0.72	25/37/37	-0.06 ±0.69	25/39/36
Tackles (%)	0.87 ± 0.07	0.80 ± 0.19	0.90 ± 0.09	-0.48 ±0.80	8/19/73	0.35 ±1.40	57/18/25
Passes (%)	0.97 ± 0.07	0.85 ± 0.22	0.96 ± 0.08	-0.63 ±0.95	7/14/79	-0.06 ±2.20	41/13/45
Average Gain/Carries (m)	1.35 ± 0.72	1.14 ± 0.67	1.22 ± 1.13	-0.27 ±1.40	28/18/53	-0.13 ±0.63	18/40/42
Effective playing time (min)	35.3 ± 8.5	32.2 ± 0.2	33.1 ± 2.5	-0.5± 0.74	6/18/76	-0.34± 0.74	12/25/63

TD: Total distance; HSR: High speed running; HMLD: High metabolic load distance; ES: Effect size; % chances: % chances that the true difference is +ive/ trivial/ -ive.

In backs and forwards, unclear differences were observed in pass and tackle success rates and average gain per carries between match 3 versus match 4 and 5 although there was a large increase in tackle success rates in match 3 vs match 5 in backs (ES: 1.20 ±0.99). In backs, there were possibly moderate and likely small increases in the frequency of passes (ES: 0.77 ±0.86 and 0.41 ±0.57 respectively) along with a possibly moderate to possibly large decrease in tackle frequency in matches 4 and 5 compared with match 3 (ES: -0.87 ±0.91 and -1.58 ±1.04 respectively). In forwards, unclear differences were observed in the frequency of tackles and carries between match 3 and matches 4 and 5. Possibly small to possibly moderate increases in passing frequency were reported in match 3 compared to matches 5 and 4 (ES: 0.41 ±0.57 and 0.77 ±0.86 respectively).

**Fig 2:**
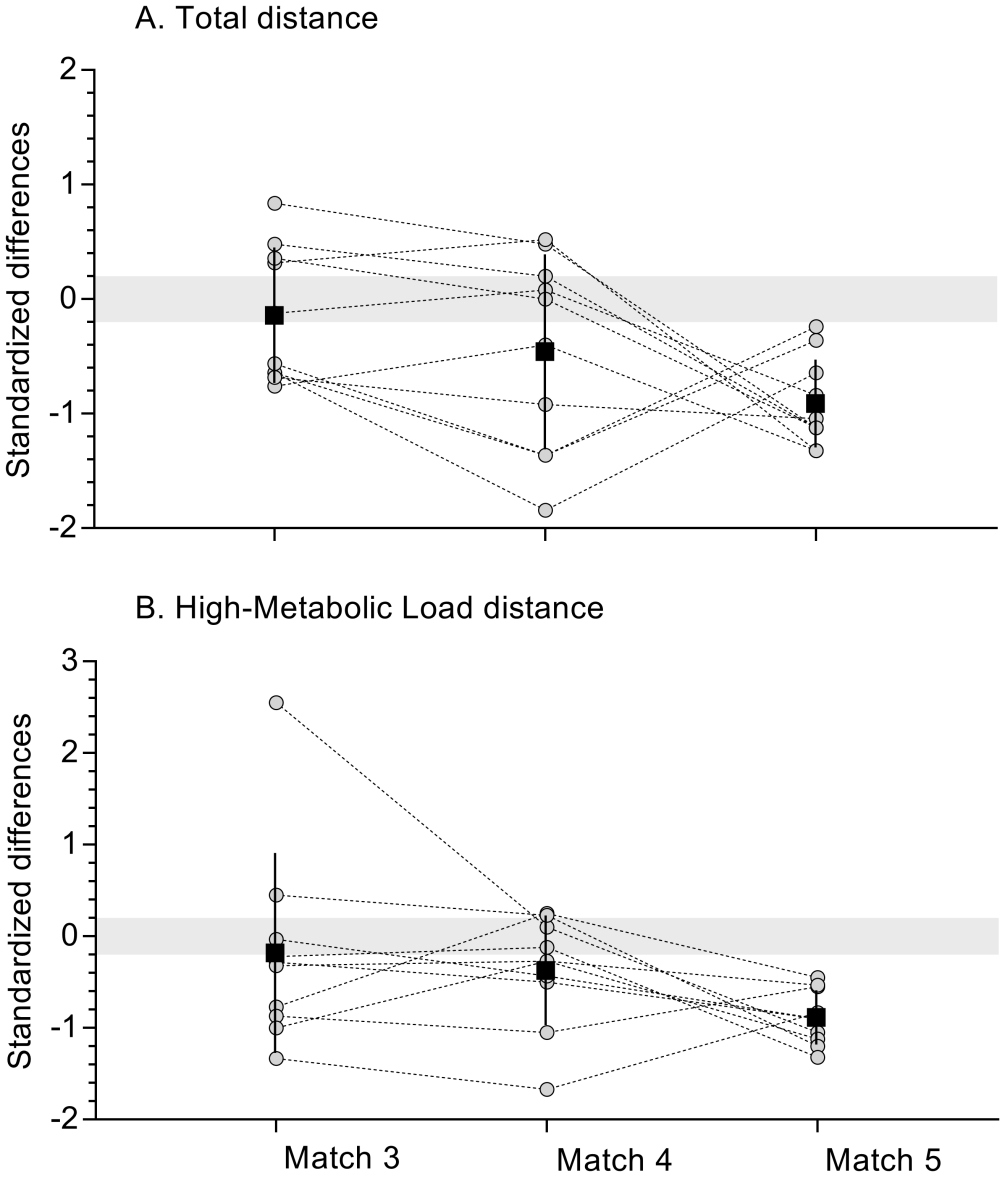
Differences in total distance covered (panel A) and high metabolic load distance covered (panel B) between the mean 10-min versus the final 10-min period, from match 3 to match 5. Grey zone stands for trivial zone (effect size ± 0.2). Grey circles: Individual observations. Black circle and bar: Mean and standard deviation.

Fig 2 reports differences in total distance covered and HMLD distance covered between the mean 10-min versus the final 10-min period, from match 3 to match 5. Small to moderate increases in total distance covered between the final 10-min and mean 10-min period were observed in matches 4 and 5 compared to match 3 (ES: 0.33 ±0.41 and 0.95 ±1.10 respectively). Regarding HMLD, there were large increases in match 4 and 5 compared to match 3 (ES: 1.25 ±0.83 and 1.24 ±1.30 respectively).

## Discussion

To our knowledge, this is the first study conducted in international junior rugby union players to investigate exposure time to match-play, running and skill-related match performance during an intensified tournament (5 matches in 19 days). The main findings were: (1) only <60% and <40% of players participated in 4 or 5 of all matches respectively despite a substantially higher availability rate for selection, (2) the two teams as a whole were able to maintain running- and skill-related performance throughout this intensive schedule, (3) in players with the highest exposure time to play, overall running performance over the final two matches was affected to a certain extent although end match running output and overall skill-related performance remained stable.

### Match exposure

In elite rugby union, the exposure time of players to competition has generally received little attention in the scientific literature [9]. No information exists for elite players in younger age categories and especially during intensified tournaments such as the u20 World Cup. In this tournament, teams are exposed to a demanding schedule of 5 matches over a 19-day period. In the present study, analysis of two international u20 teams showed that only 57% and 37% of players participated in 4 or 5 out of the 5 successive matches respectively despite player availability being nearly 90% across the tournament. These findings imply that the teams’ coaching staff recognised the need to rotate and rest players over the course of the tournament. In regards to participation in successive matches, almost two-thirds of players were exposed to 3 consecutive matches although only 22% (35% of backs and 14% of forwards) played over 60-mins in all three matches. While no information is available on the actual reasons of practitioners for selection/non-selection or substitutions of players during the present congested competition, these results again tend to suggest that rotation strategies were employed to avoid over-exposure. Similar findings have been previously identified in an elite association football club [14]. However, before any generalisations can be made additional work is necessary to determine exposure time and identify the reasons for rotation strategies across all participating teams and multiple u20 World Cup competitions.

### Overall team performance

Analyses of running and skill related performance (excluding players competing for less than 60-min) for the two teams as a whole across the 5-match schedule reported no notable changes from match to match. It is noteworthy that during the final match of the series, small to moderate increases in values were observed for the total distance covered, HMLD, number of accelerations and HMLD.Peak5-min compared to those recorded in matches 1 and 2 in both backs and forwards. The frequency of passes, successful passes and tackles and average gains per carry were lower in match 5 versus match 1 whereas the frequency of tackles and carries were higher. However, the effect sizes for these differences ranged from trivial to small. Taken together, these findings suggest that the two teams as a whole coped ‘physically’ and ‘technically’ with the demands of this intensive schedule. In the absence of similar data for rugby union, comparisons can only be made with other team sports such as soccer and rugby league. In two studies in elite soccer, neither skill nor running performance declined in two teams as a whole over several successive matches played over a short time period [22,23]. Junior rugby league players in contrast [12] reported an attenuation in overall distance run and that covered in high-speeds in the final two matches during an intensified competition (5x40-minute matches played over a 5-day period).

Several reasonable explanations may be forwarded for this lack of a reduction in match performance. First, the 4-5-day interval between matches may have been sufficient to enable full physical and/or physiological recovery and readiness for the following match [24]. Second, the systematic monitoring by the teams of recovery responses (e.g., RPE, sleep quality and quantity, muscle soreness) following competition combined with daily training load management enables evidence-based and informed decisions on player selection policies for the forthcoming match [9,25]. Third, the aforementioned standardized post-match recovery interventions possibly also aided players to maintain match performance although contrasting evidence exists for their effectiveness [26,27]. Finally, the highly developed physical qualities of players at international standards could have attenuated post-match fatigue enabling a quicker recovery. In rugby league, both the ability to perform high-intensity running and body strength are shown to minimise post-match fatigue and muscle damage markers [28]. Work in elite rugby union populations is necessary to verify this latter explanation.

### Performance in “high match exposure” players

A separate analysis of the final three matches of the competition (separated by 5-days recovery intervals) was conducted as these were considered the most demanding due to the standard of the opposition and stakes: semi-finals, finals or matches to determine team seeding in the following year’s u20 world cup. In backs who participated in a minimum 75-min play in each of these latter matches and 75% of the total team’s exposure over the entire competition, there were moderate to large decreases in total distance covered, HMLD and HMLD.Peak5min overall in games. Similar magnitude drops also occurred for HSR in forwards in matches 4 and 5 versus match 3.

These findings imply that running performance overall was negatively affected in high exposure players and might be associated with a progressive accumulation of fatigue. The decline could be associated to the cumulative perceptual, physical and physiological effects of participation in several matches over a short time frame. These results also demonstrate the importance of examining performance on an individual basis notably in players with greater exposure rather than simply for the team as a whole. It is important to note however that a reduction in effective playing time in matches 4 and 5 occurred. This drop might have partly contributed to the lower distances covered. Research to identify potential reasons for such match-to-match changes in running output related to effective playing time and other contextual factors such as score line is necessary. Similarly, simultaneous monitoring of post-match neuromuscular, blood creatine kinase, perceptual well-being, RPE and sleep responses [9] would be pertinent to complement the present external analyses of match demands. In general, work is necessary to determine the minimal time interval necessary to ensure that elite junior players are fully recovered psychologically, physically and physiologically between consecutive matches during the present tournament.

Interestingly, despite the decrease in overall running output in matches 4 and 5 versus match 3, no decrements in total distance covered or HMLD were observed during the final 10-minutes of play compared to the mean distance run for all other 10-min periods. Thus it seems that the high exposure players were able to maintain end-match running performance even at the latter end of the congested schedule. This result contrasts with previous research showing a general trend for reductions in running distances towards the end of matches in elite senior rugby union [29–31]. A reasonable explanation for this lack of a decline could be linked to players adopting a pacing strategy in order to maintain their ability to participate in key match actions throughout the entire course of play [32].

Recent research has shown that senior international rugby union players are able to maintain skill-related performance over the course of match-play even when declines in running performance occur [33]. Here, a large disparity in changes in the overall frequency and success rates of technical actions was observed in backs and forwards across the three final matches rendering difficult the interpretation of findings. For example, in match 5 compared to match 4 passing frequency improved in both playing positions whereas tackle frequency dropped in backs but increased in forwards. As these patterns might only be a reflection of the present two teams and related to the opposition teams each faced (standard, style of play, tactics), we suggest there is a need for analysis of all participating u20 teams to provide a larger sample from which more accurate conclusions can be drawn.

### Limitations and research perspectives

While two national teams collaborated on this research project, larger sample-size studies are necessary to determine exposure time and assess player rotation strategies across all participating teams and in those that are deemed to be successful or non-successful. Monitoring of the time course of various recovery markers (perceptual, physical and physiological) is also necessary to allow assessment of how a congested schedule impacts post-match recovery kinetics and subsequent readiness for play.

## Conclusions

This study shows that only <60% and <40% of players participated in 4 or 5 of all matches respectively despite high availability for selection suggesting that coaching staff operated rotation and rest strategies. It would seem that effective squad management strategies are necessary to aid junior international teams in sustaining work rate and skill proficiency over an intensified schedule as reflected in the maintaining of running and skill-related match performance by the present teams. However, in individual players reporting the highest exposure time to play especially in the most important matches (final 3 in the 5 match series), running performance over the entire match was affected to a certain extent although overall skill-related performance remained stable. Similarly, running performance during the latter stages of play was also stable. These results suggest that, while overall running performance tended to decrease in high exposure players, coaches can generally be confident in their players’ ability to maintain end-match physical- and skill-related performance even during congested schedules. This positive result might be linked to pacing and/or post-match recovery strategies and requires further investigation.

## Supporting information

S1 Data Blind Global positioning system dataset. (XLSX)
